# Methylation screening of the *TGFBI *promoter in human lung and prostate cancer by methylation-specific PCR

**DOI:** 10.1186/1471-2407-8-284

**Published:** 2008-10-03

**Authors:** Jinesh N Shah, Genze Shao, Tom K Hei, Yongliang Zhao

**Affiliations:** 1 Department of Radiation Oncology, Columbia University, New York, NY 10032, USA; 2 Center for Radiological Research, Columbia University, New York, NY 10032, USA; 3 Department of Cancer Biology, Abramson Family Cancer Research Institute, University of Pennsylvania, PA 19104, USA

## Abstract

**Background:**

Hypermethylation of the *TGFBI *promoter has been shown to correlate with decreased expression of this gene in human tumor cell lines. In this study, we optimized a methylation-specific polymerase chain reaction (MSP) method and investigated the methylation status of the *TGFBI *promoter in human lung and prostate cancer specimens.

**Methods:**

Methylation-specific primers were designed based on the methylation profiles of the *TGFBI *promoter in human tumor cell lines, and MSP conditions were optimized for accurate and efficient amplification. Genomic DNA was isolated from lung tumors and prostatectomy tissues of prostate cancer patients, bisulfite-converted, and analyzed by MSP.

**Results:**

Among 50 lung cancer samples, 44.0% (22/50) harbored methylated CpG sites in the *TGFBI *promoter. An analysis correlating gene methylation status with clinicopathological cancer features revealed that dense methylation of the *TGFBI *promoter was associated with a metastatic phenotype, with 42.9% (6/14) of metastatic lung cancer samples demonstrating dense methylation vs. only 5.6% (2/36) of primary lung cancer samples (*p *< 0.05). Similar to these lung cancer results, 82.0% (41/50) of prostate cancer samples harbored methylated CpG sites in the *TGFBI *promoter, and dense methylation of the promoter was present in 38.9% (7/18) of prostate cancer samples with the feature of locoregional invasiveness vs. only 19.4% (6/31) of prostate cancer samples without locoregional invasiveness (*p *< 0.05). Furthermore, promoter hypermethylation correlated with highly reduced expression of the *TGFBI *gene in human lung and prostate tumor cell lines.

**Conclusion:**

We successfully optimized a MSP method for the precise and efficient screening of *TGFBI *promoter methylation status. Dense methylation of the *TGFBI *promoter correlated with the extent of *TGFBI *gene silencing in tumor cell lines and was related to invasiveness of prostate tumors and metastatic status of lung cancer tumors. Thus, *TGFBI *promoter methylation can be used as a potential prognostic marker for invasiveness and metastasis in prostate and lung cancer patients, respectively.

## Background

Cancers of the lung and prostate contribute to a significant fraction of cancer-related deaths in the United States [1,2]. For lung cancer, approximately 50% of patients have metastatic disease at the time of diagnosis, which contributes to a less than 15% overall survival rate [1]. The poor survival of lung cancer patients is in part attributed to undetectable tumor micrometastasis at the time of surgery for even relatively early-stage disease, which is responsible for later relapse with the development of nodal and/or distant metastasis [3]. Furthermore, there is no highly effective curative therapy for advanced or hormone-refractory prostate cancer [4]. A better understanding of the molecular mechanisms associated with lung and prostate cancer progression may aid in the development of improved diagnosis, clinical management, and outcome prediction. In particular, the discovery of epigenetic biomarkers for cancer invasiveness and metastasis may help in the identification of patients at risk for more aggressive cancer disease courses. This would potentially help clinicians to devise effective intensified and/or novel therapeutic strategies to prevent or decrease the likelihood of tumor progression to invasiveness and metastasis in such high-risk patients.

Hypermethylation of CpG site clusters (CpG islands) within the promoter region of genes has been characterized as a common epigenetic alteration for the silencing or inactivation of tumor suppressor genes in human malignancies including lung and prostate cancers [5-7]. Due to their heritable nature, both genetic and epigenetic alterations pose a great risk for cancer development [8]. Aberrant methylation of p16INK4a, FHIT, APC, MLH1, RASSF1, CDKN2A, and DAPK has been associated with lung cancer stage, metastasis, and an increased risk of recurrence after therapy [9]. GSTP1, encoding the π-class glutathione S-transferase (GST) capable of detoxifying electrophilic and oxidant carcinogens, was the first reported gene silenced by CpG island hypermethylation in prostate cancer [10]. Subsequent studies have identified more than 40 genes that are targeted by DNA hypermethylation in prostate cancer cells, including RASSF1A (ras association domain family protein 1, isoform A), RARβ2 (retinoic acid receptor β2), p16INK4a, and PTEN (phosphatase and tensin homolog) tumor suppressor genes [11-13]. Although silencing of other tumor suppressor genes, such as RB1 (retinoblastoma-1 gene), MLH-1 (mismatch repair gene), and VHL (von Hippel-Lindau gene), through DNA hypermethylation is relatively rare in prostate cancer, it is common in other types of malignancies [5].

*TGFBI*, also known as *Betaig-h3*, is a secreted protein induced by transforming growth factor-β (TGF-β) in human adenocarcinoma cells as well as in other human cell types [14], and has been shown to possess tumor suppressor function in *in vitro *studies [15]. An earlier study from our laboratory demonstrated a dense methylation pattern of the *TGFBI *promoter in human tumor cell lines, including both lung (H522, H810, H1417) and prostate (DU145) tumor cell lines, with a complete loss or low level of *TGFBI *expression in these cell lines. In contrast, only sparsely methylated or unmethylated CpG sites were identified in cell lines with a rich level of *TGFBI *expression, including normal, immortalized, and several tumor cell lines [16]. In this study, we have examined the promoter methylation of the *TGFBI *gene in 100 cases of lung and prostate cancers by using an optimized MSP method. Our study revealed that dense *TGFBI *promoter methylation is correlated with the invasiveness of prostate cancers, and with the metastatic status of lung cancers. *TGFBI *methylation may thus serve as a useful prognostic biomarker, and our optimized MSP method may be useful for evaluation of *TGFBI *promoter methylation in a wide variety of tumor samples.

## Methods

### Lung and Prostate Cancer Patient Samples

Genomic DNA of lung cancer samples from 50 non-small cell lung cancer patients – consisting of 36 primary lung cancer tumor samples, 10 lymph node metastasis samples, and 4 distant metastasis samples – were purchased from Oncomatrix (San Marcos, CA). Frozen tissue from the prostatectomy specimens of 50 prostate adenocarcinoma patients who had undergone therapeutic prostatectomy was obtained from the Columbia University Tumor Bank. Fifteen tissue sections of seven-micron thickness were procured for each patient. A phenol-chloroform procedure was used to isolate genomic DNA from cancer specimens. For the prostate portion, we obtained approval from the Columbia IRB using "exempt status" (i.e., examination of pathological specimens that contain no patient identifier information, protocol number IRB X-10005).

In our analysis, we divided the lung cancer patients into two groups based on specimen source: patients whose tumor specimen was derived from the primary lung tumor site vs. those whose tumor specimen was obtained from a metastatic site (lymph node or distant). Lung cancer specimen source type was then correlated with *TGFBI *methylation status. Likewise, prostate cancer patients were divided into two groups based on a specific classification system: patients who did not have pathological features of locoregional invasiveness (extracapsular extension = stage pT3a, seminal vesicle involvement = stage pT3b, margin positivity, and/or regional lymph node involvement = stage pN1) vs. those who did have one or more such features of locoregional invasiveness. We then correlated the presence vs. absence of locoregional invasiveness with *TGFBI *methylation status.

As a point of clarification, we included pathological prostatectomy margin positivity as a feature that would qualify a patient's disease as having locoregional invasiveness present (in addition to pT3 or pN1 disease), which is supported by previous studies that have considered margin positivity to be a well-established feature of a more aggressive prostate cancer disease course after prostatectomy [17,18].

### Bisulfite Treatment

Using the EZ DNA Methylation Kit™ (Zymed Research, Orange, CA) protocol, 2 μg of genomic DNA from each patient sample was treated with denaturation buffer, sodium bisulfite (converting unmethylated cytosine residues to uracil), and desulphonation buffer, with elution of the bisulfite-modified DNA into 10 μl of buffer.

### *TGFBI *Regulatory Region Template Enrichment

**Figure 1:**
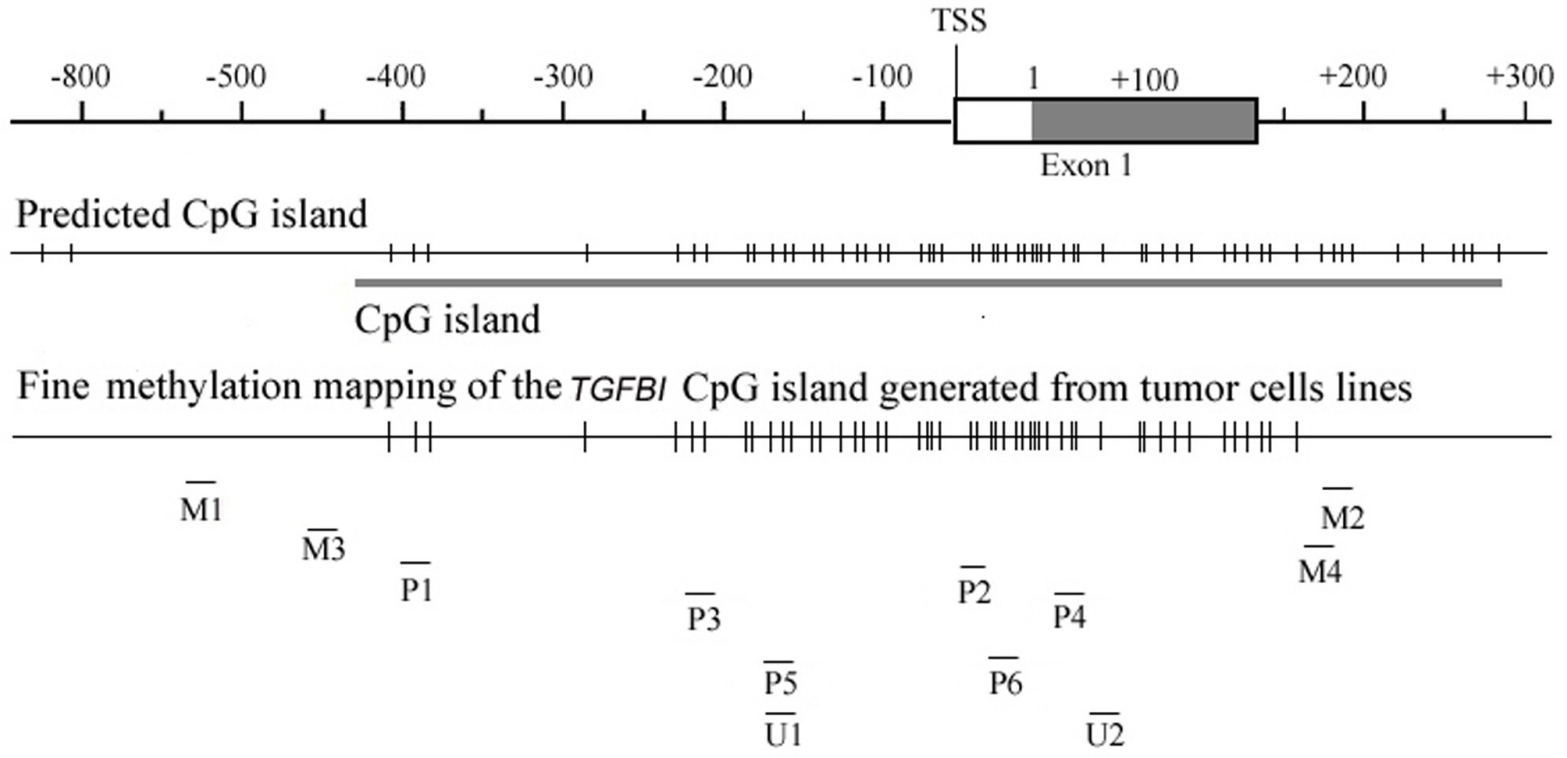
**Predicted CpG island and methylated CpG sites profile of the*****TGFBI *****regulatory region in human tumor cell lines**[**16**]. Locations of unmethylated PCR primers (M1-M4, U1-U2) and methylation-specific primers (P1-P6) are marked as "-".

To amplify the regulatory region (promoter region and first exon) of the *TGFBI *gene in each patient sample, 2 μL of the bisulfite-modified DNA was used as a template in a polymerase chain reaction (PCR) using the FailSafe™ PCR System with 2× PreMix J buffer (Epicentre Biotechnologies, Madison, WI). Primer set "M1/M2" was used in this PCR amplification. Primers M1 and M2 were designed to flank the *TGFBI *regulatory region without any potential methylation sites, thus allowing for amplification of both methylated and unmethylated DNA (i.e., primers M1 and M2 were "unbiased" vis-à-vis methylation status) (Fig. 1). The PCR conditions were as follows: a hot start at 95°C for 5 minutes (to fully denature the bisulfite-modified genomic DNA), 35 amplification cycles (94°C for 30 seconds for denaturation, 53°C for 30 seconds for primer annealing, and 72°C for 60 seconds for extension), and a final full extension at 72°C for 5 minutes. A MSP method using a methylation-specific primer set was then developed and executed using the *TGFBI *regulatory region product from the M1/M2 PCR amplification as the template for MSP, as described in the Results section.

Of note, successful template enrichment of the *TGFBI *regulatory region was demonstrated by the use of a nested "M3/M4" primer set to perform a PCR amplification on 1 μl of 1:100 diluted product from the first PCR amplification using primers M1 and M2. Primers M3 and M4 were designed to flank the *TGFBI *regulatory region, with the M3/M4 product sequence flanked by primers M1 and M2 (Fig. 1). Like primers M1/M2, M3/M4 were "unbiased" primers that bound to DNA lacking potential methylation sites so that both methylated and unmethylated DNA would be amplified. The PCR conditions for the M3/M4 reaction were the same as for the M1/M2 reaction except for use of 2× PreMix D buffer, a hot start at 94°C for 2 minutes, and 54°C as the primer annealing temperature. The intended amplification of the *TGFBI *regulatory region was proven by the presence of a 629-bp band using this M3/M4 primer pair. Thus, the PCR reaction using the M3/M4 primer pair served as a template control to ensure the successful and sequence-specific amplification of the *TGFBI *promoter, thereby mitigating the possibility of false-negative results with the MSP method. The template enrichment primer pairs were: M1:5'AGTTGGGGAGGGTGGTTAGTT3' (-548 to -529), and M2: 5'ACCCCAACTACCT AACCTTCC3' (+162 to +184), M1/M2 PCR product of 732 bp; M3: 5'TTTGTAGTGTTT TGTAGTTTTAAGATT3' (-457 to -430), and M4: 5'TAACCTTCCACAACCCCTAACCA A3' (+172 to +148), M3/M4 PCR product of 629 bp.

### Reverse Transcription and Quantitative PCR

The expression of the *TGFBI *gene was analyzed by quantitative real-time reverse transcription-PCR as described previously [16]. The first strand of cDNA was synthesized from 2 μg of total RNA using Superscript III reverse transcriptase (Invitrogen, Carlsbad, CA). Both human *TGFBI *and GAPDH RT-PCR primer sets were obtained from SuperArray Bioscience (Frederick, MD).

### Western and Northern Blotting

For analysis of TGFBI protein level, conditioned medium was collected and the protein in the medium was concentrated by adding SP sepharose at the proportion of 25 μl/ml medium (Amersham, Arlington Heights, IL) and eluted with 1× SDS loading buffer. Whole cell lyses were also collected and their protein concentrations were used to normalize the loading of medium samples. After separation and transferring to the membrane, medium samples were immunoblotted with Rabbit anti-human TGFBI polyclonal antibody (1:1,000 dilution) (R&D System, Minneapolis, MN), and the whole cell lysis samples were immunoblotted with β-actin antibody as control.

Total RNA was prepared with TRIzol reagent (Gibco, Carlsbad, CA) according to the manufacturer's instructions. For Northern blotting, 20 μg of total RNA was denatured and separated on a 1% denaturing agarose formaldehyde gel, transferred to a nylon membrane (Millipore, Bedford, MA) by downward capillary blotting in 20× SSC followed by UV crosslinking. *TGFBI *and GAPDH cDNA probes were labeled with [α-^32^P]dCTP with a random primed DNA labeling kit (Boehringer Mannheim, Mannheim, Germany). GAPDH expression level was used as control.

### Statistical Analysis

Frequencies of *TGFBI *promoter hypermethylation according to clinicopathological parameters in lung tumors, including the parameter of metastatic vs. primary lung cancer specimen, were compared using the χ^2 ^test. *P *< 0.05 was considered to be statistically significant. Methylation status of the *TGFBI *promoter in prostate cancer samples was correlated with the presence vs. absence of one or more classic pathological feature(s) of locoregional tumor invasiveness in the patients' corresponding prostatectomy specimens. Statistical comparison of locoregional invasiveness profile status (absent vs. present) according to the *TGFBI *promoter methylation status was performed using the χ^2 ^test.

## Results

### Design of Methylation-Specific Primers for the MSP Method

A 620-bp length of a CpG island, spanning the proximal promoter and the first exon of the *TGFBI *gene, was identified by screening the entire *TGFBI *genomic sequence using CpG plot prediction analysis . Our previous work had defined the methylation status of a total of 49 CpG sites across 0.6 kb of the *TGFBI *promoter in human tumor cell lines, and we had demonstrated a dense methylation pattern in lung, kidney, and DU145 prostate tumor cell lines [16]. Based on these data, in the present study we designed three pairs of methylation-specific PCR primers ("P1-P6," Fig. 1 and Table 1). Nucleotide positions were numbered relative to the translation start site (TSS) (+1). Each primer was designed to bind to a different stretch of the *TGFBI *promoter with each of these distinct stretches containing 2–3 possible methylated sites (boldface in Table 1). The nucleotide C in non-CpG sites and unmethylated CpG sites would be modified to T (sense strand) or A (antisense strand) by bisulfite treatment, whereas C would remain as C in methylated CpG sites. Thus, for example, PCR amplification with primer set P1-P2 on a particular sample would yield a PCR product only if the *TGFBI *region in this sample was actually methylated at the two CpG sites encompassed by primer P1 and the three CpG sites encompassed by primer P2. As a control, primers U1/U2 were designed to amplify unmethylated DNA, in which case the nucleotide C in CpG sites would be modified to T or A by bisulfite treatment.

**Table 1:** Primer sequences and optimized annealing temperatures for each pair of primers

Primers	Sequence	CpG sites	Location	Length (bp)	Annealing temp. (°C)
P1	5' TATGTAGGAT**C**GAAGTTTT**C **3'	2	-405 ~ -64	341	60–62
P2	5' TAAAAAC**G**CCTCC**G**CC**G **3'	3			
P3	5' GGGTATAGTG**C**GGGAG**C **3'	2	-235 ~ +22	257	65–67
P4	5' CAACC**G**CAC**G**AAAAAC**G**C 3'	3			
P5	5' GAGG**C**GTTAGG**C**GGTT**C **3'	3	-180 ~ -26	155	64.5
P6	5' AACTAAC**G**ACC**G**AC**G**AAC 3'	3			
U1	5' GAGG**T**GTTAGG**T**GGTT**T**GTT 3'	3	-180 ~ +71	252	62
U2	5' CCCACCAAAATC**A**C**A**AC**A**A 3'	3			

### Validation of Methylation-Specific Primers by MSP

To ensure the accurate and efficient amplification of methylated *TGFBI *DNA by the methylation-specific primers, we optimized the annealing temperature for these primers using 293T and H522 tumor cells as positive controls, and normal human bronchial epithelial (NHBE) cells and human mammary epithelial cells (HMEC) as negative controls. Our previous bisulfite sequencing studies demonstrated that 293T and H522 are densely methylated at the *TGFBI *promoter region, whereas NHBE and HMEC cells are unmethylated [16]. MSP was performed in a final reaction volume of 25 μl, including 1 μl of diluted PCR products (1:200) from the M1/M2 amplification as the template and 1.25 pmol of primer sets (P1/P2, P3/P4, or P5/P6). The FailSafe™ PCR System (with 2× PreMix D buffer) was used for all of the PCRs, and the PCR conditions were as follows: a hot start at 94°C for 2 minutes followed by 35 cycles of PCR (94°C for 30 seconds, annealing for 30 seconds, and 72°C for 30 seconds), and a final extension at 72°C for 5 minutes. All the PCR products were analyzed by 2% agarose gel electrophoresis and the fragments were visualized by ethidium bromide staining. The optimized annealing temperature for each primer set and the lengths of PCR products are shown in Table 1.

**Figure 2:**
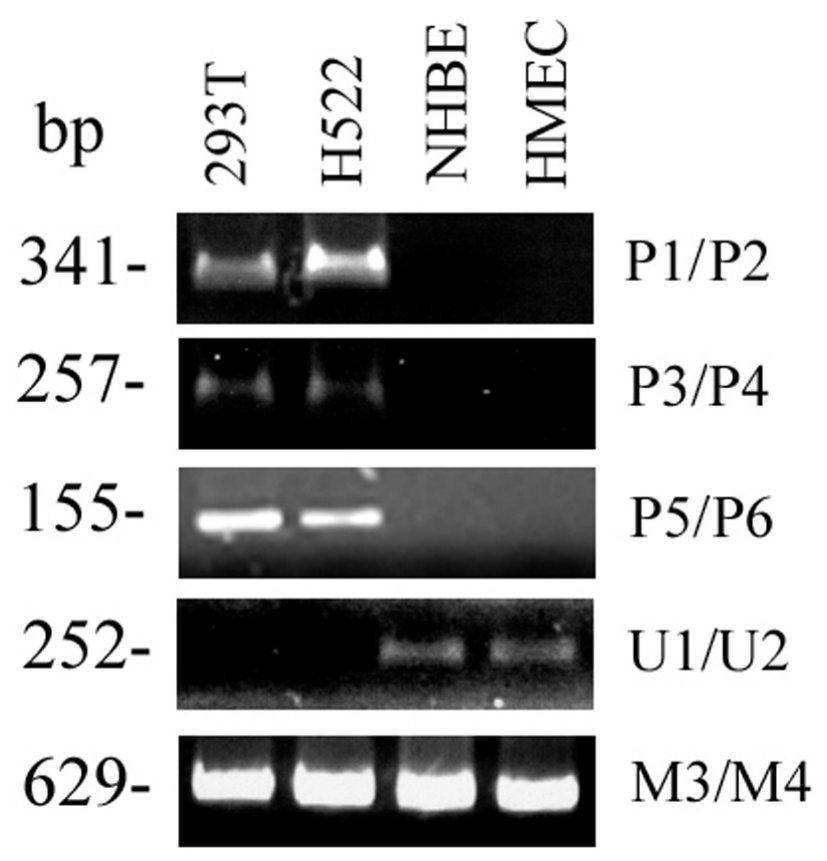
**Validation of methylation-specific primers using 293T and H522 as positive controls and NHBE and HMEC as negative controls.** U1/U2 is used for amplification of unmethylated DNA. M3/M4 is used as a *TGFBI *template control for enrichment of both methylated and unmethylated DNA templates.

Dense methylation of the *TGFBI *promoter was observed in 293T and H522 cells but not in NHBE and HMEC cells (Fig. 2). Primer set U1/U2 was used to amplify unmethylated DNA, and thus no PCR products were obtained for 293T and H522 cells but PCR products were observed for NHBE and HMEC cells.

### MSP-Based Methylation Screening of the *TGFBI *Promoter in Lung Tumor Samples

**Figure 3:**
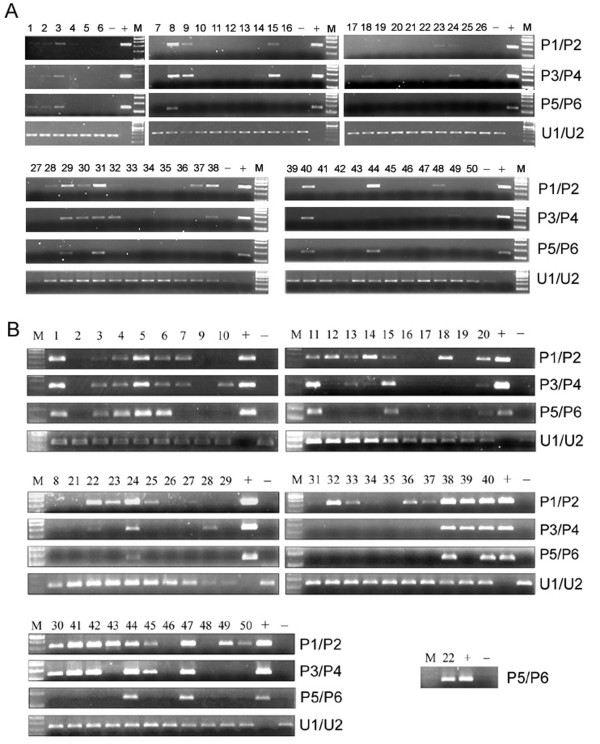
**Methylation status of the *TGFBI *promoter in lung cancer tissue samples (**A**) and prostate cancer tissue samples (**B**) using the MSP method.** "M": DNA 2-log marker; "+": positive methylated control; "-": negative unmethylated control. Numbers 1–50 correspond to each of the 50 lung and prostate cancer patients and their respective specimens.

Fifty lung cancer patient samples were screened for *TGFBI *methylation by MSP. 293T and HMEC cell lines were used as positive and negative controls, respectively. As shown in Figure 3A, 22 of 50 (44.0%) samples were identified as harboring methylated sites at different levels in the *TGFBI *promoter. Methylation frequencies detected by primer sets P1/P2, P3/P4, and P5/P6 were 20/50 (40.0%), 17/50 (34.0%), and 8/50 (16.0%), respectively. Since all samples harboring *TGFBI *methylation at the P5/P6 loci also had methylation at the P1/P2 and P3/P4 loci, methylation detected by the P5/P6 primer set indicated that at least 16 CpG sites were methylated in the *TGFBI *promoter due to the fact that the P1-P6 primers bound to locations containing a total of 16 potential methylated CpG sites (Table 1).

### MSP-Based Methylation Screening of the *TGFBI *Promoter in Prostate Tumor Samples

Fifty prostate cancer patient samples were screened for *TGFBI *methylation by MSP. H522 and HMEC cell lines were used as positive and negative controls, respectively. As shown in Figure 3B, 41 of 50 (82.0%) samples were identified as harboring methylated sites at different levels in the *TGFBI *promoter. Methylation frequencies detected by primer sets P1/P2, P3/P4, and P5/P6 were 40/50 (80.0%), 27/50 (54.0%), and 14/50 (28.0%), respectively. Consistent with the findings in lung cancer, 100% (14/14) of prostate cancer samples with methylated CpG sites detected by P5/P6 also had CpG methylation at both P1/P2 and P3/P4 regions.

### Correlation of *TGFBI *Methylation Status with Clinicopathological Features

To determine whether the *TGFBI *methylation status of the lung cancer samples was correlated with clinicopathological features of lung cancer patients, univariate analyses were carried out to correlate the methylation status of the *TGFBI *promoter with various clinicopathological parameters. Only dense methylation of the *TGFBI *promoter (i.e., 16 methylated CpG sites detected) was found to correlate with metastatic phenotype, with a significantly higher frequency of dense methylation in metastatic tumor samples (6/14, 42.9%) than in primary tumor samples (2/36, 5.6%) (*p *< 0.05) (Tables 2, 3). These results suggest a positive correlation between dense methylation of the *TGFBI *promoter and metastasis in human lung cancer.

**Table 2:** Univariate analyses of *TGFBI *methylation in non-small cell lung cancer patients

Variable	Methylation status		*P*-value
	Absent	Present	
Age (mean ± SD)	57.5 ± 8.4	60.0 ± 8.2	0.3037
Sex			0.4900
Male	26 (56.5%)	20 (43.5%)	
Female	1 (25.0%)	3 (75.0%)	
Pathological stage			0.8766
I	9 (50.0%)	9 (50.0%)	
II	6 (50.0%)	6 (50.0%)	
III	10 (62.5%)	6 (37.5%)	
IV	2 (50.0%)	2 (50.0%)	
Differentiation			0.3890
Well	1 (50.0%)	1 (50.0%)	
Moderate	15 (48.4%)	16 (51.6%)	
Poor	8 (57.1%)	6 (42.9%)	
Unknown	3 (100.0%)	0 (0.0%)	
Tumor size (cm) (mean ± SD)	4.19 ± 2.34	4.62 ± 2.62	0.6543
Tumor status			0.2946
T1	11 (61.1%)	7 (38.9%)	
T2	12 (46.2%)	14 (53.8%)	
T3	2 (66.7%)	1 (33.3%)	
T4	1 (100.0%)	0 (0.0%)	
Tx	2 (100.0%)	0 (0.0%)	
Metastatic status			0.6871
Primary tumor	22 (61.1%)	14 (38.9%)	
Lymph node metastasis	4 (40.0%)	6 (60.0%)	
Distant metastasis	2 (50.0%)	2 (50.0%)	
Histology			0.6614
Adenocarcinoma	14 (58.3%)	10 (41.7%)	
Squamous cell carcinoma	12 (52.2%)	11 (47.8%)	

**Table 3:** Methylation status of the *TGFBI *promoter in primary and metastatic (lymph node and remote) lung cancer specimens

Source of lung cancer specimen	Number of methylated CpG sites		
	
	0	5–10	16
Primary site (n = 36)	22 (61.1%)	12 (33.3%)	2 (5.6%)*
Lymph node (n = 10)	4 (40.0%)	2 (20.0%)	4 (40.0%)*
Remote metastasis (n = 4)	2 (50.0%)	0 (0.0%)	2 (50.0%)*

* *P *< 0.05

Likewise, the *TGFBI *methylation status of prostate cancer specimens was correlated with pathological features of the patients' prostatectomy specimens including those of locoregional invasiveness (i.e., extracapsular extension of tumor, seminal vesicle involvement by tumor, margin positivity with tumor, and/or regional lymph node tumor involvement) (Table 4). Similar to the findings for lung cancer samples, a significantly higher frequency of dense methylation was identified in prostate cancer specimens with the characteristic of locoregional invasiveness. Dense methylation of the *TGFBI *promoter was present in 38.9% (7/18) of prostate cancer samples with locoregional invasiveness vs. only 19.4% (6/31) of prostate cancer samples without locoregional invasiveness (Table 5, *p *< 0.05). These results suggest a positive correlation between dense methylation of the *TGFBI *promoter and locoregional invasiveness in human prostate cancer.

**Table 4:** Univariate analyses of *TGFBI *methylation in prostate cancer patients

Variable	Methylation status		*P*-value
	Absent	Present	
Gleason score			0.6241
6	2 (16.7%)	10 (83.3%)	
7 (3+4)	6 (25.0%)	18 (75.0%)	
7 (4+3)	1 (8.3%)	11 (91.7%)	
9	0 (0.0%)	1 (100.0%)	
Extracapsular extension			0.8840
Negative	8 (20.0%)	32 (80.0%)	
Positive	1 (11.1%)	8 (88.9%)	
Seminal vesicle involvement			0.5582
Negative	7 (16.7%)	35 (83.3%)	
Positive	1 (16.7%)	5 (83.3%)	
Prostatectomy margin positivity			0.4364
Negative	8 (23.5%)	26 (76.5%)	
Positive	1 (6.7%)	14 (93.3%)	
Regional lymph node involvement			0.3975
Negative	7 (18.4%)	31 (81.6%)	
Positive	0 (0.0%)	1 (100.0%)	

**Table 5:** Methylation status of the *TGFBI *promoter in prostate cancer prostatectomy specimens with or without locoregional invasiveness

Locoregional invasiveness status of prostatectomy specimen	Number of methylated CpG sites		
	
	0	5–10	16
Positive (n = 18)	2 (11.1%)	9 (50.0%)	7 (38.9%)*
Negative (n = 31)	7 (22.6%)	18 (58.1%)	6 (19.4%)*

*P < 0.05

In summary, dense methylation of the *TGFBI *promoter was associated with invasiveness in prostate cancer and metastasis in lung cancer.

### Reduced *TGFBI *Expression in Lung and Prostate Cancer Cell Lines is Mainly due to Promoter Hypermethylation

**Figure 4:**
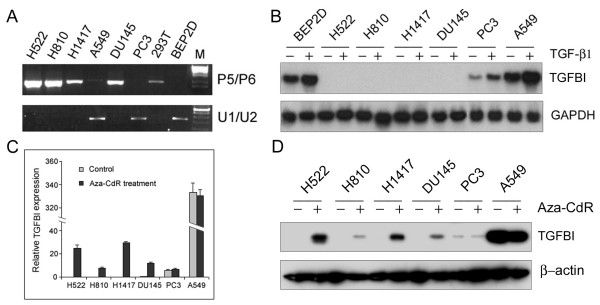
(**A**) **Methylation status of the *TGFBI *promoter in lung and prostate tumor cell lines using the P5/P6 primer pair in MSP.** (B) Induction of *TGFBI *expression in lung and prostate tumor cell lines by TGF-β1 determined by Northern blotting. Cells were treated with TGF-β1 at 5 ng/ml for 6 hours. Total RNAs were isolated and used for determining the mRNA level of the *TGFBI *gene before and after treatment. (C) *TGFBI *mRNA level in lung and prostate tumor cell lines with or without demethylating treatment determined by real-time RT-PCR. Cells were treated with Aza-CdR at 10 μM for 4 days with medium changed each day. Relative quantification of the *TGFBI *expression was performed by using real-time PCR, calibrated and normalized to its expression in DU145 cells. (D) *TGFBI *protein level in lung and prostate tumor cell lines with or without demethylating treatment examined by Western blotting.

Direct bisulfite sequencing revealed that the *TGFBI *gene promoter is hypermethylated in some human lung and prostate tumor cell lines (H522, H810, H1417, DU145), but unmethylated or sparsely methylated in A549, PC3, and BEP2D (papillomavirus-immortalized human bronchial epithelial cells) cell lines [16]. In this study, methylation status detected by the MSP method using primer pair P5/P6 was further validated in these cell lines. As expected, PCR bands were obtained for the tumor cell lines with *TGFBI *hypermethylation (H522, H810, H1417, DU145), but not for A549, PC3, and BEP2D cell lines (Fig. 4A). After treatment with TGF-β1 at 5 ng/ml for 6 h, induction of *TGFBI *mRNA level was not detectable in tumor cell lines with *TGFBI *hypermethylation, whereas A549, PC3, and BEP2D showed a high level of induction (Fig. 4B). Furthermore, treatment with a demethylating agent (Aza-CdR) at 10 μM for 4 days resulted in a substantially increased level of both *TGFBI *mRNA and protein in H522, H810, H1417, and DU145 cells, but not in A549 and PC3 cells (Fig. 4C &4D).

## Discussion

Metastasis is the major cause of death for both lung and prostate cancer patients [1-4]. Therefore, identification of biomarkers of invasiveness and metastasis would help to identify those patients who are more likely to have tumor dissemination, progression, or recurrence. We have shown previously that CpG islands of the *TGFBI *gene promoter are hypermethylated in human tumor cell lines [16]. However, the method used in our previous study was DNA sequencing of bisulfite-treated samples, which is labor-intensive and unsuitable for the rapid screening of methylation status of the *TGFBI *promoter in a large number of clinical patient samples.

Therefore, the present study was focused on optimizing and implementing a MSP method for accurate and efficient screening of *TGFBI *promoter methylation. For this purpose, we designed three pairs of methylation-specific primers based on the methylation profiles of the *TGFBI *promoter reported in human tumor cell lines [16]. MSP conditions were optimized and successfully used for examining the methylation status of the *TGFBI *promoter in 50 cases each of lung tumors and prostatectomy specimens of prostate adenocarcinoma patients. Overall, *TGFBI *methylation was present in 44.0% (22/50) of lung cancer specimens and 82.0% (41/50) of prostate cancer specimens. Interestingly, dense methylation (≥ 16 methylated CpG sites) of the *TGFBI *promoter was identified in 16.0% (8/50) of lung cancer and 28.0% (14/50) of prostate cancer specimens, respectively, which in turn correlated significantly with metastatic phenotype in lung cancers and locoregional invasiveness in prostate cancers. Therefore, cancer cells with a densely methylated *TGFBI *promoter may be prone to invasiveness in prostate cancer and metastasis in lung cancer.

It should be noted that the exact percent tumor involvement in the slices of prostate tissue used for DNA extraction was not quantified. Thus, the percentage of tumor cells in tumor specimens or proportion of tumor cells with *TGFBI *promoter methylation may be very low in some tumor samples. In the present study, we used the product from the first round of PCR (M1/M2) as template to perform the second round of MSP analysis in order to increase the amplification efficiency and avoid false-negative results.

Cell-cell adhesion and cell-substrate adhesion are critical to the preservation of normal tissue architecture, and disruption of the cell adhesion system can lead to tumor infiltration and metastasis [19]. The TGFBI protein is composed of 683 amino acids containing four homologous internal repeat domains and has been reported to function as an extracellular matrix protein that mediates cell adhesion and migration through interactions with integrin receptors α3β1, αvβ3, and αvβ5 [20-22]. TGFBI protein is a component of extracellular matrix (ECM) in lung, bladder, and skin [23], and it promotes adhesion and spreading of dermal fibroblasts and corneal epithelial cells *in vitro*. Thus, silencing of the *TGFBI *promoter by hypermethylation would result in a deficiency of the gene product, which in turn may lead to the disruption of cell-ECM interaction and contribute to invasion and metastasis of cancer cells. Our previous work identified a dense *TGFBI *methylation pattern in lung tumor cell lines (H522, H810, and H1417) and a metastasized prostate tumor cell line (DU145) [16]. Our present results showed that 16.0% of lung cancer and 28.0% of prostate cancer samples had dense *TGFBI *promoter hypermethylation and that such dense methylation was associated with metastasis in lung cancer and invasiveness in prostate cancer, suggesting a potential prognostic value of *TGFBI *hypermethylation in lung and prostate cancer patients.

The TGF-β signaling pathway plays an important role in regulating proliferation and differentiation in normal lung and prostate epithelium [24,25]. Previous reports have shown that both lung and prostate cancer cells become resistant to the antiproliferative effects of TGF-β during cancer formation [26,27]. *TGFBI*, as one of the downstream effectors of the TGF-β signaling pathway, has been shown to be expressed ubiquitously in a variety of normal human tissues but is downregulated or inactivated in many human tumor cell lines including lung and prostate tumor cell lines [15]. Thus, blockage or inactivation of the TGF-β pathway caused by loss of TGF-β receptors (I, II, III) and mutations of Smads (2, 3, 4) would lead to a lack of response to TGF-β, and subsequent downregulation of *TGFBI *expression. Our previous work showed that most CpG sites of the *TGFBI *promoter were unmethylated in normal NHBE (normal human bronchial epithelial) cells and PHEC (prostate human epithelial cells) that expressed a high level of *TGFBI *mRNA. In contrast, three lung tumor cell lines (H522, H810, and H1417) and one prostate tumor cell line (DU145) that expressed an undetectable or a much lower level of *TGFBI *mRNA showed hypermethylation in the *TGFBI *promoter [16]. Consistent with this report, in the present study MSP-based PCR products were identified in H522, H810, H1417, and DU145 tumor cells but not in A549 and PC3 cells, suggesting that the MSP method can accurately and efficiently screen the methylation status of the *TGFBI *promoter. In addition, dense methylation of the *TGFBI *promoter is responsible for gene silencing since induction of *TGFBI *expression by TGF-β1 was totally lost in the tumor cells with *TGFBI *hypermethylation, whereas demethylating treatment with Aza-CdR resulted in the re-expression of *TGFBI *in these tumor cell lines. In summary, dense methylation of the *TGFBI *promoter correlates with gene silencing that in turn may promote an advanced tumorigenic phenotype, such as invasiveness in prostate cancer and metastasis in lung cancer.

There is one report in the literature showing that overexpression of *TGFBI *promotes metastasis of SW480 colon cancer cells by enhancing extravasation [28]. However, a significant downregulation of *TGFBI *expression has been demonstrated in HT29 colon cancer cells [15] and in different types of primary human cancers [15,29-32], which is further substantiated by the present data demonstrating a correlation between promoter hypermethylation and *TGFBI *silencing in human lung and prostate cancer cells. It is highly possible that dysregulation of *TGFBI *expression is tumor cell or type-specific; moreover, *TGFBI *may be a double-edged sword whose loss or gain of expression may help promote tumorigenesis. Future studies will be required to clarify the functional complexity of *TGFBI *in human tumor progression.

## Conclusion

We designed methylation-specific primers and optimized MSP conditions to examine the methylation status of the *TGFBI *promoter in a large number of human lung and prostate cancer specimens. Our current method demonstrated that the *TGFBI *promoter was densely methylated (at least 16 methylated CpG sites) in 16.0% of lung cancer specimens and 28.0% of prostate cancer specimens, and such dense methylation correlated with metastatic phenotype in lung cancer and invasiveness in prostate cancer. In addition, *TGFBI *promoter hypermethylation was associated with gene silencing in human lung and prostate tumor cell lines. Thus, *TGFBI *promoter hypermethylation may represent a valuable prognostic biomarker in both lung and prostate cancer patients.

## Abbreviations

MSP: methylation-specific PCR; Aza-CdR: 5-Aza-2' -deoxycytidine; *RASSF1A*: ras association domain family protein 1 isoform A; *RARβ2*: retinoic acid receptor β2; *PTEN*: phosphatase and tensin homolog deleted on chromosome 10; *RB1*: retinoblastoma-1 gene; *MLH-1*: mismatch repair gene; *VHL*: von Hippel-Lindau gene.

## Competing interests

The authors declare that they have no competing interests.

## Authors' contributions

JNS and GS participated in the study design, developed the methylation-specific PCR method, analyzed the lung and prostate cancer specimens with the methylation-specific PCR method, correlated the methylation data with the clinicopathological data, performed statistical analysis, and drafted the manuscript. TKH and YZ were responsible for the study design and drafting of the manuscript and revised the manuscript critically for important intellectual content. All authors read and approved the final manuscript.

## Pre-publication history

The pre-publication history for this paper can be accessed here:



## References

[B1] Jemal A, Siegel R, Ward E, Murray T, Xu J, Thun MJ (2007). Cancer statistics, 2007. CA Cancer J Clin.

[B2] Palapattu GS, Sutcliffe S, Bastian PJ, Platz EA, De Marzo AM, Isaacs WB, Nelson WG (2005). Prostate carcinogenesis and inflammation: emerging insights. Carcinogenesis.

[B3] Kerr KM, Galler JS, Hagen JA, Laird PW, Laird-Offringa IA (2007). The role of DNA methylation in the development and progression of lung adenocarcinoma. Dis Markers.

[B4] Hegeman RB, Liu G, Wilding G, McNeel DG (2004). Newer therapies in advanced prostate cancer. Clin Prostate Cancer.

[B5] Li LC, Carroll PR, Dahiya R (2005). Epigenetic changes in prostate cancer: implication for diagnosis and treatment. J Natl Cancer Inst.

[B6] Catto JW, Azzouzi AR, Rehman I, Feeley KM, Cross SS, Amira N, Fromont G, Sibony M, Cussenot O, Meuth M, Hamdy FC (2005). Promoter hypermethylation is associated with tumor location, stage, and subsequent progression in transitional cell carcinoma. J Clin Oncol.

[B7] Hoon DS, Spugnardi M, Kuo C, Huang SK, Morton DL, Taback B (2004). Profiling epigenetic inactivation of tumor suppressor genes in tumors and plasma from cutaneous melanoma patients. Oncogene.

[B8] Jones PA, Baylin SB (2002). The fundamental role of epigenetic events in cancer. Nat Rev Genet.

[B9] Bulinski JC, McGraw TE, Gruber D, Nguyen HL, Sheetz MP (1997). Overexpression of MAP4 inhibits organelle motility and trafficking in vivo. J Cell Sci.

[B10] Lee WH, Morton RA, Epstein JI, Brooks JD, Campbell PA, Bova GS, Hsieh WS, Isaacs WB, Nelson WG (1994). Cytidine methylation of regulatory sequences near the pi-class glutathione S-transferase gene accompanies human prostatic carcinogenesis. Proc Natl Acad Sci USA.

[B11] Jarrard DF, Bova GS, Ewing CM, Pin SS, Nguyen SH, Baylin SB, Cairns P, Sidransky D, Herman JG, Isaacs WB (1997). Deletional, mutational, and methylation analyses of CDKN2 (p16/MTS1) in primary and metastatic prostate cancer. Genes Chromosomes Cancer.

[B12] Jeronimo C, Henrique R, Hoque MO, Ribeiro FR, Oliveira J, Fonseca D, Teixeira MR, Lopes C, Sidransky D (2004). Quantitative RARbeta2 hypermethylation: a promising prostate cancer marker. Clin Cancer Res.

[B13] Kuzmin I, Gillespie JW, Protopopov A, Geil L, Dreijerink K, Yang Y, Vocke CD, Duh FM, Zabarovsky E, Minna JD, Rhim JS, Emmert-Buck MR, Linehan WM, Lerman MI (2002). The RASSF1A tumor suppressor gene is inactivated in prostate tumors and suppresses growth of prostate carcinoma cells. Cancer Res.

[B14] Skonier J, Neubauer M, Madisen L, Bennett K, Plowman GD, Purchio AF (1992). cDNA cloning and sequence analysis of beta ig-h3, a novel gene induced in a human adenocarcinoma cell line after treatment with transforming growth factor-beta. DNA Cell Biol.

[B15] Zhao YL, Piao CQ, Hei TK (2002). Downregulation of Betaig-h3 gene is causally linked to tumorigenic phenotype in asbestos treated immortalized human bronchial epithelial cells. Oncogene.

[B16] Shao G, Berenguer J, Borczuk AC, Powell CA, Hei TK, Zhao Y (2006). Epigenetic inactivation of Betaig-h3 gene in human cancer cells. Cancer Res.

[B17] Bolla M, van Poppel H, Collette L, van Cangh P, Vekemans K, Da Pozzo L, de Reijke TM, Verbaeys A, Bosset JF, van Velthoven R, Maréchal JM, Scalliet P, Haustermans K, Piérart M (2005). Postoperative radiotherapy after radical prostatectomy: a randomised controlled trial (EORTC trial 22911). Lancet.

[B18] Thompson IM, Tangen CM, Paradelo J, Lucia MS, Miller G, Troyer D, Messing E, Forman J, Chin J, Swanson, Canby-Hagino E, Crawford ED (2006). Adjuvant radiotherapy for pathologically advanced prostate cancer: a randomized clinical trial. JAMA.

[B19] Stetler-Stevenson WG, Aznavoorian S, Liotta LA (1993). Tumor cell interactions with the extracellular matrix during invasion and metastasis. Annu Rev Cell Biol.

[B20] Kim JE, Jeong HW, Nam JO, Lee BH, Choi JY, Park RW, Park JY, Kim IS (2002). Identification of motifs in the fasciclin domains of the transforming growth factor-beta-induced matrix protein betaig-h3 that interact with the alphavbeta5 integrin. J Biol Chem.

[B21] Nam JO, Kim JE, Jeong HW, Lee SJ, Lee BH, Choi JY, Park RW, Park JY, Kim IS (2003). Identification of the alphavbeta3 integrin-interacting motif of betaig-h3 and its anti-angiogenic effect. J Biol Chem.

[B22] Jeong HW, Kim IS (2004). TGF-beta1 enhances betaig-h3-mediated keratinocyte cell migration through the alpha3beta1 integrin and PI3K. J Cell Biochem.

[B23] Billings PC, Herrick DJ, Kucich U, Engelsberg BN, Abrams WR, Macarak EJ, Rosenbloom J, Howard PS (2000). Extracellular matrix and nuclear localization of beta ig-h3 in human bladder smooth muscle and fibroblast cells. J Cell Biochem.

[B24] Story MT, Hopp KA, Meier DA (1996). Regulation of basic fibroblast growth factor expression by transforming growth factor beta in cultured human prostate stromal cells. Prostate.

[B25] Jetten AM, Vollberg TM, Nervi C, George MD (1990). Positive and negative regulation of proliferation and differentiation in tracheobronchial epithelial cells. Am Rev Respir Dis.

[B26] Turley RS, Finger EC, Hempel N, How T, Fields TA, Blobe GC (2007). The type III transforming growth factor-beta receptor as a novel tumor suppressor gene in prostate cancer. Cancer Res.

[B27] Teicher BA (2001). Malignant cells, directors of the malignant process: role of transforming growth factor-beta. Cancer Metastasis Reviews.

[B28] Ma C, Rong Y, Radiloff DR, Datto MB, Centeno B, Bao S, Cheng AW, Lin F, Jiang S, Yeatman TJ, Wang XF (2008). Extracellular matrix protein betaig-h3/TGFBI promotes metastasis of colon cancer by enhancing cell extravasation. Genes Dev.

[B29] Genini M, Schwalbe P, Scholl FA, Schafer BW (1996). Isolation of genes differentially expressed in human primary myoblasts and embryonal rhabdomyosarcoma. Int J Cancer.

[B30] Zhao Y, El-Gabry M, Hei TK (2006). Loss of Betaig-h3 protein is frequent in primary lung carcinoma and related to tumorigenic phenotype in lung cancer cells. Mol Carcinog.

[B31] Calaf GM, Echiburu-Chau C, Zhao YL, Hei TK (2008). BigH3 protein expression as a marker for breast cancer. Int J Mol Med.

[B32] Becker J, Volland S, Noskova I, Schramm A, Schweigerer LL, Wilting J (2008). Keratoepithelin reverts the suppression of tissue factor pathway inhibitor 2 by MYCN in human neuroblastoma: a mechanism to inhibit invasion. Int J Oncol.

